# Impact of growth hormone on scoliosis

**DOI:** 10.1002/pdi3.26

**Published:** 2023-09-26

**Authors:** Xin‐Kai Zhang, Xiang Li, Ming‐Yan Shi, Man Zhang, Pei‐Kang Wang, Hai‐Lun Yao, Xin Tan, Xiang Yu, Yu‐Fei Shao, Xing Liu

**Affiliations:** ^1^ Department of Orthopedic in Children's Hospital of Chongqing Medical University National Clinical Research Center for Child Health and Disorders Ministry of Education Key Laboratory of Child Development and Disorders Chongqing China

**Keywords:** growth hormone, scoliosis, short stature

## Abstract

Growth hormone (GH) therapy has been used in patients with growth retardation and short stature due to various reasons. The safety of GH treatment is still debatable, one of which is the occurrence and development of scoliosis. In order to explore the effects of GH on the occurrence or progression of scoliosis, this review summarizes previous studies, summarizes the influence of GH treatment on the pathogenesis and progression of scoliosis, and analyzes the possible mechanism of GH in the pathogenesis and progression of scoliosis. Hopefully, it can provide a direction for future research.

## 1 SCOLIOSIS

Scoliosis is a three‐dimensional spine deformity involving rotation of the vertebra and scoliosis. Scoliosis affects 2%–5.2% of children and adolescents^1^, ^2^ and can be divided by age into infant scoliosis (0–3 years), juvenile scoliosis (4–9 years), and adolescent scoliosis (>10 years). Its causes are varied and may be related to genetics, environment, hormones, metabolism, and neuromuscular development.^3^, ^4^, ^5^ They are classified into congenital scoliosis, neuromuscular scoliosis, syndrome‐associated scoliosis, and idiopathic scoliosis, of which adolescent idiopathic scoliosis (AIS) is the most common. Children can be manifested as unequal shoulder height, torso asymmetry, and the back of a knife. There may be a significant deformity in the progression of lateral curvature during the rapid growth period, leading to waist pain, adverse psychological reactions, long‐term low quality of life, and even cardiopulmonary function impairment.^6^ The imaging findings showed that the Cobb angle was greater than 10° in the coronal plane. The Cobb angle is the angle formed by the two steepest vertebrae in the curve, the upper boundary of the upper vertebrae and the lower boundary of the lower vertebrae, and is the most commonly used clinically to assess the severity of scoliosis.

The specific mechanism of the occurrence and progression of scoliosis needs to be further studied. The etiological theories of AIS can be classified into gene correlation theory, hormone and metabolism theory, biomechanical theory, bone marrow mesenchymal stem cell theory, neural theory, and environmental theory. Among them, hormone and metabolism is one of the hot topics in current research studies. Growth hormone (GH), sex hormones, melatonin, leptin, and other hormones are involved in bone metabolism. Currently, many studies have elaborated on the relationship between hormone‐related abnormalities and abnormal bone metabolism in AIS patients from various perspectives and the possible mechanism. The decrease in bone density caused by hormone‐related abnormalities may be one of the core contents of the pathogenesis of AIS. Histomorphological studies showed that compared with unaffected individuals, bone volume fraction, trabecular bone thickness, and bone cell and osteoblast density were decreased in AIS patients.^7^ In addition, studies have found that the risk of developing idiopathic scoliosis is significantly correlated with the duration of rapid linear bone growth, especially with the peak rate of spinal growth.^8^, ^9^, ^10^, ^11^ Secondly, the ability of bone growth and development is represented by bone maturity. In general, the lower the bone maturity, the greater the bone growth capacity and the likelihood of the development and progression of scoliosis; conversely, the higher the bone maturity, the less likely the occurrence and progression of scoliosis. Bone age, Risser staging, and Tanner–Whitehouse‐III RUS score are now used to assess bone maturity.^12^, ^13^ Third, the severity of idiopathic scoliosis appears to be higher in patients who are overweight or obese when it first appears. In a retrospective cohort study of 150^14^ (≥10 years old) patients with spinal asymmetry, BMI ≥85 percentile was associated with a greater mean Cobb angle and increased risk of Cobb angle ≥40°. Therefore, hormone secretion and metabolism in the body play an important role in scoliosis.

## 2 GROWTH HORMONE

In the 1920s, GHs were discovered to control growth. Human GH consists of 191 amino acid residues. GH is secreted by the pituitary gland rhythmically,^15^ and GH secretion increases significantly after sleep. The effect of GH reaches its peak in adolescence. The effect of GH on the body can be divided into acute effects and long‐term effects. The effect of GH on human metabolism is an immediate effect, which can be realized within a few minutes. GH can promote amino acid transport into cells, promote lipolysis, and increase blood sugar. In addition, the GH can promote the secretion of thymosin and stimulate B lymphocytes to produce antibodies to improve the activity of NK cells and macrophages, so GH is involved in regulating the immune system. GH can promote the growth of almost all tissues and organs, especially bone, muscle, and internal organs, which is the long‐term effect of GH. GH promotes the increase in the size and number of cells in most body organs by promoting the proliferation of bone, cartilage, muscle, and other tissue cells and increasing the synthesis of proteins in the cells. For bone growth, GH directly stimulates the differentiation of growth plate chondrocytes, widens the epiphyseal plate, deposits bone matrix, and promotes the longitudinal growth of bone. GH deficiency reduces bone formation, leads to growth retardation and low bone mass in children, and increases the risk of fracture in adults.^16^

It is worth noting that the secretion of GH is regulated by other hormones, mainly the dual regulation of hypothalamic GH‐releasing hormone (GHRH) and somatostatin. Meanwhile, other hormones such as sex steroids, glucagon, insulin, parathyroid hormone (PTH), and glucocorticoid can also promote the secretion of GH.^16^, ^17^ PTH, estrogen, and insulin promote GH secretion,^18^, ^19^, ^20^ whereas glucocorticoids and glucagon inhibit GH secretion.^21^ Studies have shown estrogen can activate and promote GH secretion and IGF‐1 expression through ER.^22^ Therefore, estrogen may affect bone growth and development through the GH‐IGf axis. Estrogen and androgen levels and GH/IGF‐1 protein expression peak during puberty. However, GH/IGF‐1 protein levels return to preadolescent levels in adulthood. This trend is consistent with the rapid development of AIS during adolescence, suggesting that the GH/IGF‐1 axis may play an essential role in AIS.^19^, ^23^, ^24^

**FIGURE 1:**
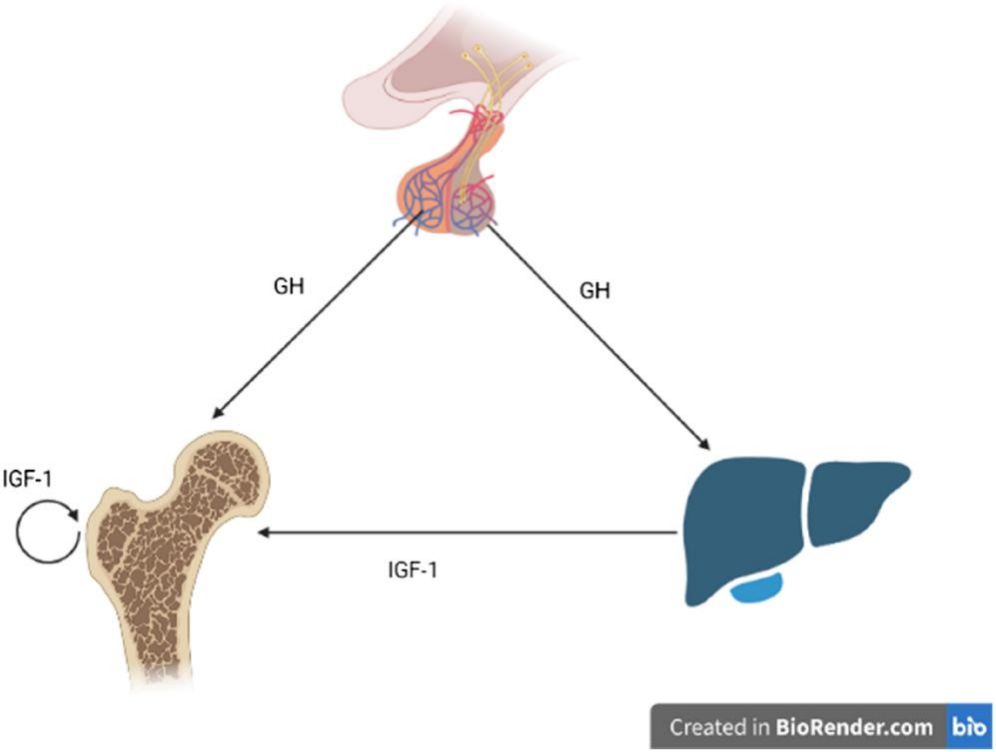
GH/IGF‐1 axis: Growth hormone (GH) is secreted by pituitary somatotrophs in a pulsatile manner and acts on peripheral tissues, either directly or indirectly, through the stimulation of insulin‐like growth factor 1 (IGF‐1) synthesis and secretion (The figure was created in BioRender.com).

After the GH release, the primary mechanism of action is to stimulate the synthesis and release of IGF in peripheral tissues and the liver. These liver factors bind to circulating IGF‐binding proteins and travel through the bloodstream to multiple target tissues, most importantly bone tissues^25^, ^26^ (Figure 1). GH can directly stimulate the prechondrocytes or germinal layer cells of the epiphyseal growth plate to differentiate into chondrocytes, induce the expression of IGF‐1 gene, and act on the IGF‐1 receptor of osteoblasts through autocrine or paracrine mode to promote the proliferation and hypertrophy of chondrocytes and transform them into osteocytes, thus promoting the growth of bones. Results of animal models^27^, ^28^ showed that paracrine IGF‐1 mainly affected bone trabecule, whereas endocrine IGF‐1 mainly affected the bone cortex. In mice with removed estrogen receptor alpha (ERα), decreased IGF‐1 protein resulted in slower bone growth.^29^, ^30^ Clinical studies^31^ have shown that excess GH and consequent elevation of serum IGF‐1, as in patients with acromegaly, are associated with increased bone turnover. IGF‐1 is essential for the differentiation of osteoclasts (OCL) and the maintenance of OCL function.^32^ In general, GHs and IGFs aid in bone formation and mineral acquisition, but in excess, they can stimulate bone resorption and impair bone remodeling.

Therefore, IGF‐1 is an essential mediator in GH regulation of growth. The production of IGF‐1 depends on the GH. However, studies have shown that the GH is released in a pulse‐type manner with significant daily fluctuation. IGF‐1 and IGFBP3 can indirectly reflect the GH secretion in healthy children.^33^ As the main protein of IGF‐1 transport, IGFBP3 can prolong the IGF‐1 cycling time and stabilize the serum IGF‐1 concentration. Studies have shown that IGFBP3 deficiency leads to decreased bone volume fraction and enhances osteoclast differentiation, which is detrimental to bone growth.^34^ Previous results of our research group^35^ showed that the level of serum IGFBP3 in children with AIS was significantly lower than that in the control group, and the difference was statistically significant. Decreased serum IGF‐1 levels affect linear bone growth and radial bone growth, an essential determinant of bone mechanical performance. Therefore, it is speculated that decreased IGFBP3 levels may lead to decreased bone volume fraction and bone density in AIS patients, and abnormal GH/IGF axis may be a potential pathogenesis of AIS.

## 3 THE INCIDENCE AND PROGRESSION OF SCOLIOSIS UNDER rhGH TREATMENT

Recombinant human growth hormone (rhGH) is a replacement therapy through the exogenous synthesis of GH, which can avoid the serious complication of human growth hormone (hGH). rhGH is currently being used for a variety of reasons since 1985,^36^ including growth hormone deficiency (GHD), idiopathic short stature (ISS), growth retards caused by Turner syndrome (TS), Prader–Willi syndrome (PWS), chronic renal insufficiency, small for gestational age (SGA), Noonan syndrome (NS), mucopolysaccharidosis (MPS),^37^ chronic renal failure, SHOX gene mutation,^38^ Silver–Russell syndrome (SRS)^39^ and other diseases. There are many patients with scoliosis or progression of original scoliosis, among which scoliosis is the most common in patients with PWS and TS.^40^, ^41^ In addition, studies have reported that the incidence of scoliosis in patients treated with GH after organ transplantation is higher than that in patients not treated with GH, and the incidence is statistically significant.^42^ Significant questions are whether GH contributes to the development and progression of scoliosis and how it affects the occurrence and progression of scoliosis.

GH treatment has been shown to increase overall, lumbar and femoral neck bone mineral density (BMD Z score), reduce body fat percentage, and improve peak bone mass accumulation.^43^ In one study, 36%–38% of AIS girls met the definition of osteopenia, which occurs when an individual has a z‐score of −1 or lower. This systemic osteopenia is seen in axial skeletal sites such as the hip and spine and around the distal tibia and radius.^44^, ^45^ Low bone mass in AIS affects cortical and trabecular compartments and is associated with abnormal bone mineralization, bone morphology, trabecular microstructure, volumetric bone density, overall bone mass, and mechanical strength.^46^

The National Cooperative Growth Study (NCGS) and Kabi International Growth Study (KIGS) were the two large‐scale, multicenter, pharmaco‐epidemiological studies on patients receiving GH replacement therapy. The resulting publications recorded many adverse reactions in patients receiving GH therapy. However, the incidence of these complications was rare and not always proven to be caused by GH therapy. Wang et al. showed a 4% incidence of scoliosis in children treated with GH, similar to the 2.2% found in another study of healthy school‐age children.^47^ However, a later study by the Growth Hormone Research Institute showed that GH treatment accelerated growth, exacerbating scoliosis.^48^ Similarly, in 2010, the National Cooperative Growth Study (NCGS) reported 238 cases (of 54,996 patients treated with GHs), of which 76 had prior progression of scoliosis.^49^ In addition, certain pathological conditions themselves increase the risk of scoliosis, such as Prader–Willi syndrome (PWS) and Turner syndrome (TS).^39^, ^41^, ^50^

### 3.1 Growth hormone deficiency

Children with GHD present with early severe growth retardation, delayed bone age, concentrated fat distribution, low blood concentrations of GH, insulin‐like growth factor 1 (IGF‐1), and insulin‐like growth factor binding protein 3 (IGFBP3).^51^ A multistep examination is needed to make a definitive diagnosis. GH therapy was first used in children with GHD,^52^ the best indication for GH therapy. A study from the KIGS database showed that patients with GH deficiency had the highest rates of GH therapy, and patients with IGHD or congenital GHD had a lower frequency of both causal and treatment‐related adverse reactions when treated with GH. For scoliosis, the correlation of treatment^53^ was 0.2%. Scoliosis has not been the focus of observation in GHD patients treated with GH.

### 3.2 Idiopathic short stature

Idiopathic short stature (ISS) is an excluded diagnosis based on short stature (height less than 2 SDS^54^ below the average height for the same age, sex, and population) without a systemic, genetic, or endocrine diagnosis associated with short stature.^55^, ^56^ In a sample size of 2450 children treated with rhGH for ISS, treatment‐emergent events (TEAE) reported scoliosis in 1.6%.^57^ Se‐Jun Park et al. conducted a retrospective study.^58^ They divided 1128 ISS patients treated with rhGH into two groups. 1093 patients had no scoliosis before treatment and 40 (3.7%) developed new scoliosis during treatment after an average of 2 years of rhGH treatment; the incidence was similar to that reported for a control general population without rhGH treatment.^59^, ^60^ In addition, 67 children had scoliosis before treatment. After treatment, 11 cases (16.4%) of scoliosis progressed.

The study showed that age and sex were statistically significant factors in the incidence of scoliosis. The mean age of newborn scoliosis patients during GH treatment was nearly 2 years older than that of patients without scoliosis. In addition, earlier studies reported a higher incidence of scoliosis in girls than in boys.^61^ When the curve is 10°, the male‐to‐female ratio is equal, but when the angle is greater than 30°, the male‐to‐female ratio increases to 10:1.^62^ Predicted sex ratios range from 5:1 to 7:1.^63^ However, it is common knowledge that idiopathic scoliosis is more prevalent in women than men. Univariate analysis showed statistically significant increases in age, bone age, sex, and annual height. However, the two groups had no statistical difference in the duration or type of rhGH treatment.

It was found that age at scoliosis, bone age at the start of rhGH treatment, and duration of rhGH treatment were significant factors associated with the progression of scoliosis. In previous studies, the rate of progression of scoliosis in idiopathic scoliosis patients who were not treated with rhGH varied from 10% to 70%. Among 727 idiopathic scoliosis patients who were not treated with rhGH, Lonstein and Carlson reported that the rate of scoliosis progression was approximately 23.2% over an average of 14 months of follow‐up. Trobisch et al. reported that in adolescents with scoliosis who were not treated with rhGH, the progression rate was about 10%–20% when the Cobb angle was less than 20° and more than 70% when the Cobb angle was greater than 20°.^64^ Scoliosis progression is rare if the Cobb's angle is <30° and the patient has reached bone maturity.^65^ Accurately predicting the progression of scoliosis depends on the clinician's experience, especially the ability to interpret the remaining growth potential.

To investigate the progression of scoliosis during GH treatment, Yun, Y. et al.^66^ carried out a retrospective study that analyzed radiographic changes in the spine of ISS patients treated with GH and a control group of idiopathic scoliosis patients of the same age who were regularly followed up. They measured the following indicators, which were scoliosis Cobb's angle, apical vertebral translation, coronal balance, and pelvic obliquity. The results showed that the progression of Cobb's angle and apical translation in the rhGH group was more obvious than in the control group. Cobb's angle increased by 1° per year and apical translation increased by 1.2 mm per year in the rhGH treatment group.

### 3.3 Prader–Willi syndrome

Spinal deformity is a significant problem in patients with PWS. The prevalence of PWS scoliosis is high, ranging from 30% before the age of 10 years to 80% after the age of 10 years (13–15 years), similar to Grimberg, A. et al.'s report of a 37.5%–86% incidence of scoliosis in the PWS population. The incidence of much higher idiopathic scoliosis in the population is 1.5%–1.7%.^67^ Grootjen, L. N, et al.'s^68^ were one of the few groups to conduct a long‐term trial, and their study included only preschoolers, not adolescents, during the trial period. This avoids the effects of other hormonal changes in the body during puberty. They conducted an 8‐year randomized controlled clinical trial and found that 8 years of GH treatment had no adverse effect on the prevalence and severity of scoliosis in children under 11 years of age with PWS. Moreover, it was found that the apparent density of bone minerals BMADLS SDS was negatively correlated with the Cobb angle, suggesting that optimizing BMD status in children with PWS is crucial.

Interestingly, the progression of scoliosis during GH therapy was also associated with lower paravertebral muscle volume as measured by CT, and the paravertebral muscle growth rate in patients with aggravated scoliosis was significantly lower than that in patients without recombination or scoliosis. And the age of the former is greater than the latter two.^67^ GH and IGF‐1 have been shown to increase muscle mass in patients suffering from various diseases related to muscle atrophy.^69^ The result suggests that muscle growth may prevent scoliosis progression. This raises the possibility that GH‐mediated increases in muscle mass and strength may reduce the occurrence or progression of scoliosis, but currently there is no data to support this effect in patients with PWS.^70^, ^71^, ^72^ Studies have shown that GH improves the height, body composition, fat percentage and distribution, and other metabolic markers in children with PWS. Preliminary reports of improvements in cognitive development during GH therapy have emerged. Taken together, these studies suggested that it was important to monitor scoliosis in this high‐risk population with or without treatment with rhGH and that rhGH does not significantly increase the risk of developing scoliosis.

### 3.4 Turner syndrome

TS is characterized by gonadal hypoplasia (87%–96%), short stature (88%–100%), and many phenotypic abnormalities, including skeletal deformities, the most common sex chromosome abnormality in women. Scoliosis is relatively common in TS patients (12%–28%). If mild cases are included, the prevalence of all TS cases increases to 59%.^73^ The study of Ricotti, S et al.^74^ found that growth had a significant impact on scoliosis occurring in patients with TS. That is, the average age of onset of scoliosis in TS patients is higher than that in the normal population, so it is presumed that the onset age of TS patients is delayed due to growth retardation. But the study was unable to find evidence that GH promotes the onset and progression of scoliosis. In addition, many other studies have found a higher incidence of scoliosis in patients with TS and suspected that the GH may contribute to the onset and progression of scoliosis.^40^, ^49^, ^75^ Therefore, clinical practice should pay attention to the growth and development of children. If scoliosis has developed, drug withdrawal should be considered, and active intervention should be taken to prevent further progress of scoliosis. Kim et al.^46^ recorded 11.6% (43 patients) of TS patients with scoliosis, while 2.4% of women of similar age had idiopathic scoliosis; however, two of five patients with TS do not receive GH. In NCGS, the incidence of scoliosis in TS patients was 0.6%,^32^ whereas that in non‐TS patients was 0.39%.^76^ Of the 36 cases of scoliosis documented by NCGS with TS, 16 were progressed, and the rest were new cases or unknown histories. Thus, TS itself increases the risk of scoliosis, and GHs appear to increase the progression of existing scoliosis.^50^, ^76^

### 3.5 Other diseases

Noonan syndrome (NS) is a common hereditary multisystem disorder that includes a range of clinical features, including short stature, facial features, congenital heart disease, and bone abnormalities.^77^ Scoliosis is one of the NS skeletal abnormalities. Romano et al.^78^ reported 6 cases of scoliosis in 370 patients over a mean period of 5.6 years of rhGH treatment. The GH does not seem to promote the occurrence and progression of scoliosis in NS patients.^79^ For NS patients, the possibility of other comorbidities being considered during GH therapy is superior to the occurrence of scoliosis.

Mucopolysaccharide accumulation syndrome (MPS), a group of lysosomal accumulation diseases, is a series of clinical symptoms caused by lysosomal hydrolytic enzyme defects. At present, there are no clear conclusions about the occurrence and progression of scoliosis in patients with MPS treated with GH. Previous studies have found a tendency to promote scoliosis, and MPS patients may have a certain tolerance to rhGH. At present, there are no clear conclusions about the occurrence and progression of scoliosis in patients with MPS treated with GH. Previous studies have found a tendency to promote the progression of scoliosis, and MPS patients may have a certain tolerance to rhGH.^80^

Children who undergo organ transplantation frequently develop growth abnormalities, which point to the use of rhGH treatment.^81^ The occurrence of scoliosis after solid organ transplantation in children and adolescents was studied. Helenius et al. analyzed 196 children who survived solid organ transplantation such as the kidney, liver, and heart.^48^ Scoliosis occurred in 20 (37%) of 54 patients treated with GH and in 23 (16%) of patients not treated with GH (*p* = 0.0016). In multiple logistic regression analysis, rhGH treatment was an important independent risk factor for postoperative scoliosis.^51^

## 4 CONCLUDING REMARKS

The following conclusions can be drawn from the current studies on the effect of rhGH treatment on the incidence and progression of scoliosis. First, the incidence of scoliosis in patients with idiopathic short stature treated with recombinant human GH during treatment was not significantly different from that in the general population. Second, recent studies have found that GH therapy may promote the progression of scoliosis in ISS patients with scoliosis. Third, patients with the syndrome, especially PWS and TS patients, have a higher incidence and progression of scoliosis than that of the general population. Currently, more scholars believe that the use of GH will not increase the incidence and progression of scoliosis in this population, and its high incidence and progression are more likely to be due to the impact of its own underlying disease, which requires close monitoring of spinal growth.

In addition, the mechanism of action of the GH on scoliosis is unclear and requires further study. The current study suggests many possible causes for the onset and progression of scoliosis due to GH treatment. By comparing the possible factors of AIS with the action of GH in vivo, we found that the GH may induce the onset and development of AIS in promoting the growth rate. Growth acceleration may amplify biomechanical abnormalities if the patient has a skeletal deformity or if paravertebral muscle asymmetry causes biomechanical abnormalities in the patient. More prospective studies are needed in this regard. In addition, excess GH can activate osteoclasts and increase bone resorption, which is common in patients with acromegaly. In some cases, high doses of GH are associated with significantly elevated serum IGF‐1 levels and symptoms of GH overload (ankle swelling or hip pain),^38^ which have been reported. However, it is not known whether it affects the spine. Studies^1^, ^49^, ^82^ suggest that the potential risk of scoliosis is due to rapid bone growth and immature development rather than a direct effect of GHs.

Despite the potential orthopedic complications associated with hGH treatment, GH benefits bone and skeletal components and may outweigh these problems. The benefits of GH therapy for patients with PWS outweigh the risks and suggest that scoliosis during GH therapy is not an indication to discontinue the drug. The question of whether to discontinue GH therapy and the timing of discontinuation when scoliosis or scoliosis progression occurs during GH therapy is worthy of consideration in addition to whether muscle strength training during GH therapy may prevent the onset and progression of scoliosis may also be of interest. In conclusion, the exact mechanism of the effect of GH on scoliosis is unknown, and further research is needed.

## AUTHOR CONTRIBUTIONS

Xin‐Kai Zhang drafted the original manuscript, and revised it. Ming‐Yan Shi, Xiang Li, Man Zhang and Pei‐Kang Wang collected literature and conceptualized. Hai‐Lun Yao and Xin Tan conducted clinical investigations. Xiang Yu, Yu‐Fei Shao designed figure and table. Professor Xing Liu is accountable for all aspects of the work in ensuring that questions related to the accuracy or integrity of any part of the work are appropriately investigated and resolved. Each author fulfills related requirements and accepts the final submission.

## CONFLICT OF INTEREST STATEMENT

The authors declare that the research was conducted in the absence of any commercial or financial relationships that could be construed as a potential conflict of interest.

## ETHICS STATEMENT

Not applicable.

## Data Availability

Data sharing is not applicable to this article as no new data were created or analyzed in this study.
